# Dyslipidemia in HIV-positive patients: a randomized, controlled, prospective study on ezetimibe+fenofibrate versus pravastatin monotherapy

**DOI:** 10.7448/IAS.17.1.19004

**Published:** 2014-08-21

**Authors:** Anna M Grandi, Eleonora Nicolini, Laura Rizzi, Sara Caputo, Filippo Annoni, Anna M Cremona, Chiara Marchesi, Luigina Guasti, Andrea M Maresca, Paolo Grossi

**Affiliations:** 1 Department of Clinical and Experimental Medicine, University of Insubria, Varese, Italy; 2 Department of Internal Medicine, Ospedale di Circolo, Varese, Italy; 3 Department of Surgical and Morphological Sciences, University of Insubria, Varese, Italy

**Keywords:** HIV infection, protease inhibitors, dyslipidemia, pravastatin, ezetimibe, fenofibrate

## Abstract

**Introduction:**

We designed a randomized, controlled prospective study aimed at comparing efficacy and tolerability of ezetimibe+fenofibrate treatment versus pravastatin monotherapy in dyslipidemic HIV-positive (HIV+) patients treated with protease inhibitors (PIs).

**Methods:**

We consecutively enrolled 42 HIV+ dyslipidemic patients on stable PIs therapy (LDL cholesterol >130 mg/dl or triglycerides 200–500 mg/dl with non-HDL cholesterol >160 mg/dl). After basal evaluation, patients were randomized to a six-month treatment with ezetimibe 10 mg/day+fenofibrate 200 mg/day or with pravastatin 40 mg/day. Both at the basal evaluation and after the six-month treatment, the patients underwent blood tests for lipid parameters, and muscle and liver enzymes.

**Results:**

At baseline, the two groups (21 patients each) were similar with regards to gender, age, BMI, blood pressure and virologic and metabolic parameters. After the six-month therapy, total cholesterol, LDL cholesterol and non-HDL cholesterol decreased significantly (*p*<0.01) in both groups. high-density lipoprotein (HDL) cholesterol increased (44±10 to 53±12 mg/dl, *p*<0.005) and triglycerides decreased (from 265±118 mg/dl to 149±37 mg/dl, *p*<0.001) in the ezetimibe+fenofibrate group, whereas both parameters remained unchanged in the pravastatin group. Mean values of creatine kinase (CK), alanine aminotransferase and aspartate aminotransferase were unchanged in both groups; only one patient in the pravastatin group stopped the treatment after two months, due to increased CK.

**Conclusions:**

In dyslipidemic HIV+ patients on PI therapy, the association of ezetimibe+fenofibrate is more effective than pravastatin monotherapy in improving lipid profile and is also well tolerated.

## Introduction

The introduction of highly active antiretroviral therapy (HAART) has fundamentally changed the natural history of HIV disease, leading to a significant increase in life expectancy. However, a wide range of metabolic alterations has been found with increased frequency in HIV-positive (HIV+) patients treated with HAART, especially during therapy with protease inhibitors (PIs). These abnormalities mainly involve lipids (hypertriglyceridemia, high levels of low-density lipoprotein (LDL) cholesterol, low levels of high-density lipoprotein (HDL) cholesterol) and glucose metabolism (insulin resistance, hyperinsulinemia, fasting hyperglycaemia, impaired glucose tolerance) [1–6] and play a relevant role in increasing cardiovascular morbidity and mortality in the affected patients [7–11].

The following approach has been outlined for the treatment of dyslipidemia in HIV+ patients [12]: statin monotherapy if LDL cholesterol is >130 mg/dl or the triglycerides level is between 200 and 500 mg/dl with non-HDL cholesterol >160 mg/dl, fibrate monotherapy if triglycerides level is >500 mg/dl. Since many statins have a high likelihood of interacting with antiretroviral drugs (mainly through an action on cytochrome P-450), the treatment should only include those statins with the least potential for drug interaction; examples of such statins are pravastatin and fluvastatin, which are relatively less potent lipid-lowering agents [12]. Fibrates do not significantly interact with HAART and are effective in decreasing triglycerides; however, they exert a very small effect on LDL cholesterol. Single drug treatment in HIV+ patients on HAART often fails to meet target lipid goals [13–15], also because statins do not significantly control hypertriglyceridemia. In these cases, guidelines suggest a statin+fibrate treatment; however, in HIV+ patients the risk of muscle and liver toxicity during statin or statin+fibrate treatment is significantly higher than in the general population [12].

Ezetimibe is a lipid-lowering agent that inhibits the intestinal absorption of cholesterol [16] and is characterized by a cytochrome P-450-independent metabolism. Both efficacy and safety of ezetimibe monotherapy as well as of ezetimibe+statin coadministration have been widely demonstrated in dyslipidemic non-HIV patients [16–22]: ezetimibe monotherapy reduced LDL cholesterol by 17–20% compared with placebo, triglycerides levels also decreased significantly by 5% and HDL cholesterol increased by 2–3%. Furthermore, studies on HIV+ patients showed that ezetimibe is as effective as statin monotherapy in decreasing cholesterol, is well tolerated and does not interact with HAART [23–25].

Starting from these data, we decided to evaluate whether ezetimibe+fibrate can be a useful and safe alternative to statin monotherapy in dyslipidemic HIV+ patients treated with PIs. To this aim, we designed a randomized, controlled, prospective, open pilot study, comparing the lipid-lowering efficacy and the tolerability of a six-month treatment with ezetimibe+fenofibrate versus pravastatin monotherapy.

## Methods

### Patients

Among the HIV+ patients referred to our outpatients’ clinic of Infectious Diseases, we consecutively enrolled HIV+ adults (age>18 years) with the following characteristics: stable therapy with PIs for at least 12 months, LDL cholesterol >130 mg/dl or triglycerides 200–500 mg/dl with non-HDL cholesterol >160 mg/dl, unresponsive to diet and regular physical exercise for at least three months.

Exclusion criteria were as follows: history of dyslipidemia before antiretroviral therapy, history of cardiovascular and/or cerebrovascular diseases, Cushing's syndrome, hypothyroidism, type 1 or type 2 diabetes mellitus, renal failure, previous or current therapy with lipid-lowering agents, antihypertensive drugs or oestrogens, current abuse of drugs and/or alcohol.

Following these criteria, we enrolled 42 patients (35 men, mean age 46±7 years, weight 73.5±11.4 kg, BMI 25.2±3 kg/m^2^).

The Ethical Committee of the Ospedale di Circolo approved the study and all the patients gave their informed consent.

### Study design

This is a pilot study, therefore we chose a sample of convenience since we lacked the data for a reliable sample size power analysis.

At the basal evaluation, each patient underwent a physical examination, clinic blood pressure measurement (three measures by sphygmomanometer, at 5-minute intervals) and blood tests for total cholesterol, HDL cholesterol, LDL cholesterol, triglycerides, aspartate aminotransferase (AST), alanine aminotransferase (ALT), creatinine phosphokinase (CK), serum creatinine, fasting glucose, fasting insulin, high-sensitivity C-reactive protein (hsCRP), CD4+ count and viral load.

The patients were then randomized to a six-month treatment with ezetimibe 10 mg/day+fenofibrate 200 mg/day or with pravastatin 40 mg/day.

Throughout the treatment, patients underwent monthly visits, with clinical evaluation and blood tests for plasma creatinine, CK, ALT and AST.

After six months, the patients underwent the last evaluation with blood tests for total cholesterol, HDL cholesterol, LDL cholesterol, triglycerides, CK, ALT, AST, serum creatinine, fasting glucose, fasting insulin, hsCRP, CD4+ count and viral load (same data gathered for the basal evaluation).

LDL cholesterol was measured directly on serum. HOMA index, a parameter of insulin resistance, was calculated as fasting plasma insulin (pmol/l)×fasting plasma glucose (mmol/l)/22.5 [26]. Insulin concentration was measured by an antibody method with a solid-phase 125I radioimmunoassay (Coat-A-Count insulin, Diagnostic Products Corp). This method has a sensitivity of 6.6 pmol/l and a coefficient of variation of 7.1% at insulin values of 6–240 pmol/l.

### Main safety measurements

At each visit:

1. if ALT and/or AST were ≥3 times the upper normal limit (confirmed by a second test within seven days), the patient was excluded from the study;
2. if CK was ≥5 times the upper normal limit (confirmed by a second test within seven days) with or without myalgia, muscle weakness and/or cramps, the patient was excluded from the study.

### Statistical analysis

The statistical evaluation of the results was carried out using 2 (between two treatment groups) by 2 (repeated measures with two levels: basal and six-month evaluation) analysis of variance (ANOVA), followed by the test of Scheffé, in order to:

1. compare mean basal and six-month values between the two groups
2. evaluate the effects of the two treatments (ezetimibe+fenofibrate vs. pravastatin) on metabolic parameters.

The distribution of PIs and NRTIs in the two groups was compared by means of χ^2^ test.

The data are expressed as mean±SD; a probability value <0.05 was considered statistically significant.

## Results and discussion

At the basal evaluation, the two groups were not significantly different with regard to gender (men/women 17/4 vs. 18/3, ns), age (45±9 vs. 47±7 years, ns), body mass index (Table 1), clinic systolic (121±12 vs. 123±14 mmHg, ns) and diastolic (83±8 vs. 81±10 mmHg, ns) blood pressure, hsCRP, duration of HIV infection (13±7.4 vs. 14.7±7.6 years, ns) and CD4+ count (Table 1). Both duration of HAART therapy (8±6.2 vs. 6±5.3 years, ns) and distribution of different PIs and NRTIs (Table 2) were similar between the two groups. Viral load was undetectable (<75 copies/mm^3^) in 39 patients (95%), only two patients in the pravastatin group had detectable viral load (one patient 130 copies/mm^3^ and the other 100 copies/mm^3^).

**Table 1:** Mean values (±SD) of parameters at the basal evaluation and after the six-month treatment

	Pravastatin (n = 20)		Ezetimibe + Fenofibrate (n = 21)		ANOVA	
	
	Basal	6-month	Basal	6-month	Time	Treatment
BMI, kg/m^2^	25.4±3.1	25.2±3.6	24.9±3.1	25.1±2.8	0.64	0.72
Total cholesterol, mg/dl	248±39	224±43*	241±34	199±35*^,^°	0.007	0.012
HDL cholesterol, mg/dl	47±11	46±9	44±10	53±12*^,^°	0.41	0.008
LDL cholesterol, mg/dl	149±32	125±36*	149±33	122±37*	0.005	0.59
Non-HDL cholesterol, mg/dl	200±35	178±38*	196±36	147±32*^,^°	0.005	0.011
Triglycerides, mg/dl	263±96	248±89	265±118	149±37*^,^°	0.38	<0.001
Creatinine, mg/dl	0.86±0.12	0.88±0.14	0.89±0.16	0.85±0.14	0.52	0.47
Fasting glucose, mg/dl	83±12	85±14	87±12	84±9	0.61	0.58
HOMA index	3.9±2.6	4.2±3.2	4.1±2.8	4.3±3.5	0.37	0.55
ALT, U/l	45±37	39±28	34±22	36±20	0.49	0.51
AST, U/l	44±38	39±34	41±29	37±25	0.53	0.46
CK, U/l	159±116	158±119	186±125	190±128	0.72	0.43
hsCRP, mg/L	3.13±3.25	2.44±1.40	2.88±3.02	2.41±1.29	0.23	0.52
CD4+ count, n/mm^3^	598±238	582±241	605±320	592±285	0.61	0.73

BMI: body mass index; HDL: high-density lipoprotein; LDL: low-density lipoprotein; HOMA: homeostasis model assessment; ALT: alanine aminotransferase; AST: aspartate aminotransferase; CK: creatine kinase; hsCRP: high-sensitivity C-reactive protein; ns: non-significant.

ANOVA Time: basal versus 6 months, Treatment: ezetimibe + fenofibrate vs. pravastatin.

* 0.02 < *p*<0.001 6-month vs. basal evaluation.

° 0.05 < *p*<0.001 ezetimibe + fenofibrate vs pravastatin at 6-month.

**Table 2:** Distribution of different PIs and NRTIs in the study population

	Pravastatin (20)	Ezetimibe+Fenofibrate (21)	*p*
Fosamprenavir* n (%)	2 (10)	4 (19.1)	ns
Atazanavir* n (%)	9 (45)	8 (38.1)	ns
Saquinavir* n (%)	3 (15)	2 (9.5)	ns
Darunavir* n (%)	2 (10)	3 (14.3)	ns
Lopinavir/ritonavir* n (%)	3 (15)	4 (19)	ns
Tipranavir* n (%)	1 (5)	0	ns
Zidovudin° n (%)	3(15)	5(23.8)	ns
Tenovovir° n (%)	5(25)	6 (28.6)	ns
Abacavir° n (%)	5(25)	5 (23.8)	ns

* PIs: protease inhibitors.

° NRTIs: nucleoside reverse transcriptase inhibitors.

ns: non-significant.

Mean values of all the metabolic parameters were not significantly different between the two groups (Table 1).

At the two-month visit, one patient treated with pravastatin stopped the treatment because of increased CK level (two blood tests one week apart); CK level returned within the normal limit after three weeks.

All the other 41 patients completed the six-month treatment; HAART therapy remained unchanged in all patients during the study****. Body weight and diet also did not change.

After the therapy, mean values of body mass index, serum creatinine, fasting glucose, HOMA index, ALT, AST, CK, CD4+ count (Table 1) and viral load were unchanged in both groups. Mean values of hsCRP, marker of systemic inflammation, decreased in both groups, but the change was not statistically significant (Table 1).

Total cholesterol, LDL cholesterol and non-HDL cholesterol decreased significantly in both groups (Table 1); mean percentage decreases of both total cholesterol (−6.4±9.5% vs. −10.5±9.1%, *p*<0.05) and non-HDL cholesterol (−22.8±11.2% vs. −12.6±8.8%, *p*=0.003) were significantly greater in the ezetimibe+fenofibrate group, whereas the extent of mean percentage LDL reduction (−17.9±9.8% vs. −16.6±8.5%, ns) was similar between the two groups.

HDL cholesterol increased and triglycerides decreased significantly in the ezetimibe+fenofibrate group, whereas both parameters remained unchanged in the pravastatin group (Table 1).

ANOVA (2×2 factors) showed a significant treatment effect of ezetimibe+fenofibrate on lipid parameters (Table 1).

To our knowledge, this is the first study testing efficacy and feasibility of a new therapeutic strategy for dyslipidemia in HIV+ patients on HAART therapy. Our results show that a six-month treatment with ezetimibe+fenofibrate is well tolerated and is as effective as pravastatin in reducing LDL cholesterol, while being also able to reduce triglycerides and to increase HDL cholesterol, both instead unchanged with pravastatin monotherapy.

Since we wanted to compare the effects of the two treatments on lipid profile and on liver and muscle enzymes, we tried to avoid possible confounding factors by selecting patients with normal lipid profile before HAART treatment, on stable therapy with boosted PIs for at least 12 months and with at least three months of diet and physical exercise ineffective on dyslipidemia. In order to avoid any influence on lipid profile, we excluded patients with previous or on-going treatment with antihypertensive drugs, statins or oestrogens; we also excluded subjects with current alcohol and/or drug abuse, both able to modify liver and muscle enzymes. We decided that blinded treatment was not necessary since the placebo effect was in our opinion negligible with regards to the parameters evaluated in the study. Finally, we did not rely on the Friedewald equation to calculate LDL level, but we chose to measure directly LDL cholesterol on serum.

Patients were selected based on the 2003 guidelines which suggest pharmacological treatment of dyslipidemia in HIV+ patients when LDL cholesterol is >130 mg/dl or triglycerides level is between 200 and 500 mg/dl with non-HDL cholesterol >160 mg/dl. For these lipid levels, the guidelines suggest statin monotherapy. We opted for pravastatin because, while its antidyslipidemic efficacy is lower than other statins, it is well tolerated in HIV+ subjects and it does not significantly interact with anti-retroviral drugs since it is not metabolized by cytochrome P450 CYP3A4 [14].

Due to the difficulties in reaching lipid goals in HIV+ patients, therapeutics options other than statin monotherapy have to be taken into consideration. In clinical practice, fibrates are added to statin therapy when triglycerides are very high or, more often, when statin monotherapy fails to achieve target lipid goals. The fibrate+statin combination is included in the guidelines for the general population and also for HIV+ patients, but this treatment needs to be closely monitored because of the increased risk of side effects. The risk is even more relevant in HIV+ patients on HAART due to the known greater incidence of liver and muscle damages in such patients [12]. Therefore, we decided to test the combination of fibrate with ezetimibe that inhibits Niemann–Pick C1 Like 1 protein, thus reducing the intestinal absorption of dietary and biliary cholesterol [16].

In our prospective, randomized, controlled study, ezetimibe+fibrate treatment induced a decrease of LDL cholesterol similar to that observed in pravastatin monotherapy; this is in agreement with a limited sample of data in HIV+ patients showing that ezetimibe monotherapy is as effective as pravastatin in decreasing LDL cholesterol [24,25]. The very significant decrease of triglycerides observed in our patients was due to fibrate action; it should however be noted that some studies on non-HIV patients found a limited, but statistically significant decrease of triglycerides during ezetimibe monotherapy [19,20]. We also observed a significant increase in HDL cholesterol, which has already been described during ezetimibe monotherapy in studies on non-HIV patients; this HDL increase has been connected to the greater increase in concentration of apolipoprotein A-I with ezetimibe compared to placebo [19–22].

With regards to the evaluation of our results, we want to underline that HAART, body weight and diet, which are all possible confounding factors, remained unchanged in all the patients.

Both treatments did not significantly influence insulin sensitivity as HOMA index was unchanged after the six-month therapy. It is interesting to note that basal mean value of HOMA index was above the upper normal limit (>2.5) [27] in both groups, indicating that most patients were insulin resistant (16 pts in the pravastatin group and 18 in the ezetimibe+fibrate group). We cannot rule out the possibility that the complete lack of changes in glucose parameters in both groups is due to the small size of our sample; however, we note that the possible negative effect of statin therapy on glucose homeostasis has not been established yet.

With regards to systemic inflammation, basal mean values of hsCRP were similar between the two groups and showed a non-statistically significant decrease in both groups as well. It should however be noted that the extent of CRP reduction was greater in the pravastatin arm than in the ezetimibe+fenofibrate arm. The anti-inflammatory effect of statin is well known; hence, it cannot be excluded that a similar study on a larger number of patients would show a significantly different trend of CRP in the two treatment groups.

With regards to the possible side effects of the two lipid-lowering treatments, mean levels of liver enzymes and CK remained unchanged in both groups; only one patient treated with pravastatin had to stop therapy after two months because of very high levels of CK that returned within the normal limits after three weeks from the discontinuation of the treatment.

The main limitation of our study is the small number of patients enrolled, which is due to the restrictive selection criteria employed. However, we believe that the finding of statistically significant differences between the two treatments in a small number of subjects makes the results of our study even more interesting. After the beginning of our study, other statins that are more potent than pravastatin and without clinically significant interactions became available (e.g. rosuvastatin); it is possible that our results would be different if the comparison were to be made with one of the new statins. Finally, this study was designed to compare the two treatments’ effects on biochemical endpoints; hence, our analysis cannot give insights about the effectiveness of the treatments in reducing cardiovascular diseases; this endpoint would require a different, multi-year study on a much larger number of patients.

## Conclusions

In HIV+ patients on HAART, it is essential to implement therapies, such as treatment of dyslipidemia, aimed at reducing cardiovascular risk. Our pilot study shows that in these patients the association of ezetimibe+fenofibrate is more effective than pravastatin monotherapy in improving lipid profile and is also well tolerated.
